# Mystery of the lost gallstone‐Part 2

**DOI:** 10.1002/ccr3.3368

**Published:** 2020-09-21

**Authors:** Konstantinos A. Boulas, Aikaterini Paraskeva, Alexandros Triantafyllidis, Maria Nathanailidou, Konstantinos Chatzipourganis, Anestis Hatzigeorgiadis

**Affiliations:** ^1^ Department of General Surgery General Hospital of Drama Drama Greece

**Keywords:** cholecystectomy, gallstones, laparascopy, obstruction, perforation, small bowel

## Abstract

If gallbladder perforation occurs during cholecystectomy, every spilled gallstone should be retrieved to minimize possible late gallstone‐related complications.

## Graphical abstract

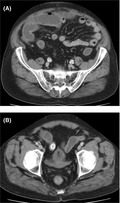

An otherwise‐healthy 81‐year‐old male patient, with a history of open cholecystectomy for acute cholecystitis 7 years ago, presented to the emergency department with clinical, laboratory, imaging findings suggestive of complete distal small‐bowel obstruction. CT revealed (a) predominantly dilated proximal small bowel loops with the transition point in the terminal ileum at the right iliac fossa; (b) an intraperitoneal large 3 * 4 cm calcified stone at the upper right side of the Douglas pouch (Figure 1A and B). Early surgery performed due to high possibility for strangulation.

**FIGURE 1:**
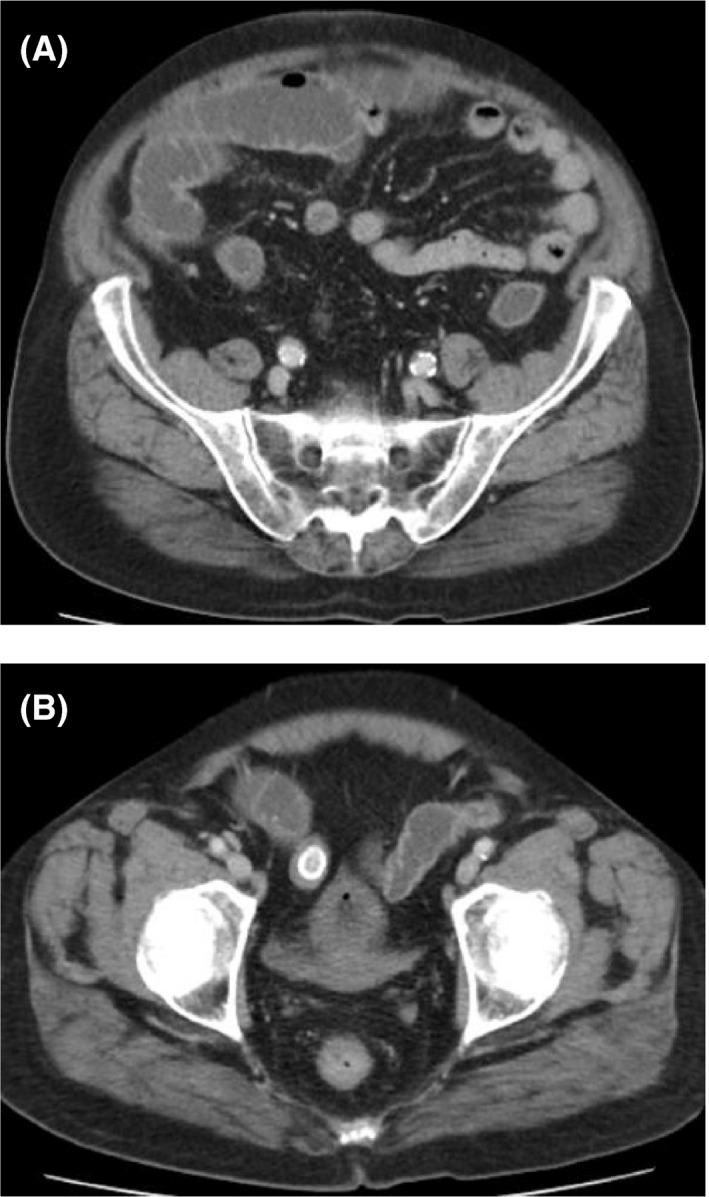
A and B, CT revealed the presence of (A) predominantly dilated proximal loops with the transition point in the terminal ileum at the right iliac fossa; (B) an intraperitoneal large 3 * 4 cm calcified stone at the upper right‐side of the Douglas pouch at the entrance of the minor pelvis

## 1 QUIZ QUESTION: WHAT IS YOUR DIAGNOSIS?

Laparotomy revealed rotation of an ileus loop around adhesive bands between greater omentum and parietal peritoneum at the right iliac fossa. After adhesiolysis, a large round‐shaped stone retrieved from the right side of the minor pelvis entrance. Taken into account history of cholecystectomy, the stone considered to be unretrieved gallstone. The sequence of events was as follows: Gallbladder perforation during cholecystectomy resulted in spillage of gallstones; an unretrieved gallstone migrated due to extensive irrigation and gravity to minor pelvis causing phlegmon formation^1^; although self‐limited, inflammation resulted in adhesions formation which eventually led to bowel obstruction. Although criteria for gallstone‐ileus are not present, a form of gallstone‐ileus is the diagnosis as bowel obstruction should be considered as late complication related to unretrieved gallstones.^2^

## CONFLICT OF INTEREST

The authors declare that they have no conflict of interests.

## AUTHOR CONTRIBUTIONS

All authors equally accessed the data and contributed to the preparation of the manuscript. BA and HA: were equally responsible for making and performing treatment decisions. HA: reviewed the manuscript for critical intellectual content and had the final approval.

## INFORMED CONSENT

Informed consent was obtained from the patient.

## STATEMENT OF HUMAN AND ANIMAL RIGHTS

The present article does not contain any studies with human or animal subjects performed by any of the authors.
